# How emotional labor leads to turnover intention: the mediating role of job burnout and the moderating role of social support among rural physical education teachers in China

**DOI:** 10.3389/fpsyg.2025.1596750

**Published:** 2025-06-10

**Authors:** Pu Sun, Lifang Wang, Xi Chen, Mingyue Cui, Ke Ma, Ling Yan

**Affiliations:** ^1^Department of Psychology and Education, Capital University of Physical Education and Sports, Beijing, China; ^2^Department of Student Affairs, Hexi University, Zhangye, China; ^3^Department of Physical Education, Capital University of Physical Education and Sports, Beijing, China

**Keywords:** emotional labor, job burnout, turnover intention, social support, rural physical education teachers

## Abstract

**Objective:**

To examine the mediating role of job burnout in the relationship between emotional labor strategies and turnover intention among rural physical education (PE) teachers in China, and to explore the moderating effect of social support in this mechanism.

**Methods:**

A cross-sectional survey was conducted among 1,761 rural PE teachers using validated self-report scales. Structural equation modeling (SEM) via AMOS 26.0 was used to test the mediating effects of job burnout, followed by moderated mediation analyses using PROCESS macro Model 7, controlling for gender, age, teaching tenure, and education level.

**Results:**

Job burnout significantly mediated the associations between all three emotional labor strategies and turnover intention. Surface acting showed both direct and indirect effects, while deep acting and genuine expression were associated with turnover intention only through burnout. Additionally, social support moderated the relationship between surface acting and burnout, but not for the other two strategies.

**Conclusion:**

Emotional labor strategies influence turnover intention both directly and indirectly through job burnout, while social support serves as a key buffering resource against the negative impact of surface acting on burnout. These findings underscore the importance of optimizing emotional labor management strategies and strengthening social support networks to enhance teacher wellbeing and promote educational equity in rural schools.

## Introduction

In the field of education, emotional labor plays a vital role in shaping teaching quality, student outcomes, and teachers’ professional wellbeing (Hargreaves, 2000; Yin et al., 2019). Emotional labor refers to the regulation and expression of emotions in accordance with organizational or societal expectations to maintain a desirable emotional state (Hochschild, 2012). For teachers, emotional labor goes beyond classroom management; it is a core professional skill that fosters a positive learning environment and supports students’ holistic development (Horner et al., 2020). However, the prolonged demands of emotional regulation may lead to the depletion of psychological resources, resulting in job burnout and heightened turnover intention, which negatively impact teacher health and educational quality.

Previous research has highlighted that teachers’ emotional labor varies significantly across educational contexts (Kariou et al., 2021). Although urban teachers also experience emotional demands, they are often supported by more robust institutional structures and career development opportunities, which help buffer the negative effects. In contrast, rural teachers—particularly physical education (PE) teachers—are situated in resource-constrained and marginalized environments, making them more vulnerable to the chronic stress associated with intensive emotional labor (Zhang et al., 2025).

Rural PE teachers, as a distinct occupational group, face multiple emotional labor challenges. Due to unequal resource allocation and the long-term marginalization of PE as a subject, these teachers often lack professional identity, have limited career advancement opportunities, and receive insufficient social support (Shen et al., 2024). In addition, they are frequently required to take on administrative and interdisciplinary teaching tasks beyond their core responsibilities, which further increases their emotional burden. Although the Chinese government has recently promoted the integration of urban and rural compulsory education and introduced several policy initiatives—such as the *Opinions on Promoting Integrated Reform and Development of Urban and Rural Compulsory Education within Counties* and the *Guidelines on Strengthening the Construction of the Rural Teaching Workforce in the New Era*—structural inequality and weak support systems remain persistent challenges, directly affecting the occupational stability and psychological wellbeing of rural teachers.

While previous studies have examined the relationship between emotional labor and job burnout, there is a lack of systematic research on rural PE teachers—a group often overlooked in the literature. Compared to their peers, these teachers not only bear higher emotional labor burdens but also operate within chronically under-resourced educational systems (Li et al., 2020). There is an urgent need to identify the mechanisms linking their professional stressors to potential support pathways. To address this gap, the present study employs Conservation of Resources (COR) theory to investigate how emotional labor influences turnover intention through job burnout and to examine the moderating role of social support (Grandey and Melloy, 2017). According to COR theory, resource depletion intensifies the negative effects of emotional labor, while social support, as a critical external resource, can buffer and restore psychological capacity.

Furthermore, this study situates the emotional labor challenges of rural PE teachers within the framework of Sustainable Development Goal 4 (SDG 4), addressing the dual imperatives of educational equity and teacher wellbeing. By elucidating the mechanisms of emotional labor under resource-constrained conditions, this study contributes to the theoretical intersection of educational justice and psychological health, while also offering practical strategies to improve rural teacher support systems and reduce teacher attrition—findings with broader relevance for other developing countries.

## Theoretical framework and hypotheses

### Understanding emotional labor through the lens of conservation of resources theory

This study adopts the Conservation of Resources (COR) theory as its primary theoretical framework and integrates the strategy-based classification from emotional labor theory to examine how emotional labor affects job burnout and turnover intention among rural PE teachers in resource-constrained settings. According to COR theory, individuals strive to conserve, acquire, and protect valued resources such as psychological energy, self-efficacy, and social support (Hobfoll, 1989). When individuals experience resource loss or a significant imbalance between investment and return, they encounter sustained stress. Without timely replenishment, this may lead to resource exhaustion, which manifests as emotional fatigue, reduced efficacy, and interpersonal withdrawal—core components of job burnout (Hobfoll et al., 2018).

Within educational systems, emotional labor is an often overlooked but critical mechanism of resource consumption. It requires teachers to regulate and express emotions in alignment with institutional expectations, functioning as a form of psychological labor embedded in daily instruction and interpersonal interactions (Hochschild, 2012). Grandey and Gabriel (2015) categorize emotional labor into three strategies: surface acting, deep acting, and genuine expression.

Surface acting involves modifying outward emotional displays while suppressing inner feelings. This incongruence generates emotional dissonance and, in resource-poor contexts, heightens emotional exhaustion and burnout (Brotheridge and Lee, 2002). For rural PE teachers, surface acting is especially common. In order to maintain classroom discipline or fulfill additional administrative duties, they often suppress negative emotions and conform to institutional norms, thereby accelerating the depletion of emotional resources.

Deep acting, by contrast, entails cognitive and emotional reframing to align internal emotions with external expressions. Although it may reduce emotional dissonance, deep acting relies heavily on emotional regulation capacity and cognitive resources. Under inadequate support conditions, long-term deep acting may also lead to overconsumption of internal resources (Wang et al., 2019).

Genuine expression—where external emotional displays are consistent with inner feelings—is the least resource-intensive strategy. It is typically associated with better mental health and higher job satisfaction (Çetin and Çolak, 2020). However, its implementation depends on teachers’ professional identity and a sense of organizational security—conditions that are often unmet in rural schools with limited support structures.

Within the COR framework, social support is conceptualized as a vital external resource that enhances resilience and buffers against resource depletion (Cohen and Wills, 1985). Surface acting, which demands the most unreciprocated emotional output, is particularly sensitive to support mechanisms. Without adequate social support, teachers may experience a negative cycle of unchecked resource loss. Thus, this study focuses on the moderating effect of social support in the pathway from surface acting to job burnout. Prior research has shown that emotional support from administrators (e.g., empathy and affirmation) and instrumental support from colleagues (e.g., shared workload) can effectively mitigate the stress reactions caused by surface acting, thereby reducing the risk of emotional exhaustion and burnout (Gabriel et al., 2015; Moon et al., 2014).

Although recent studies have examined the relationship between emotional labor and burnout, most have focused on urban teachers or professionals in health care and service industries. There remains a critical lack of research on rural PE teachers—a group characterized by extreme resource scarcity and diverse role demands. These teachers face a persistent structural dilemma of high emotional investment and low emotional return, which compromises both their occupational stability and psychological health. Addressing this research gap, the present study employs COR theory to construct an integrated model linking emotional labor strategies with job burnout and turnover intention, while examining the buffering effect of social support as a key external resource.

**Figure 1:**
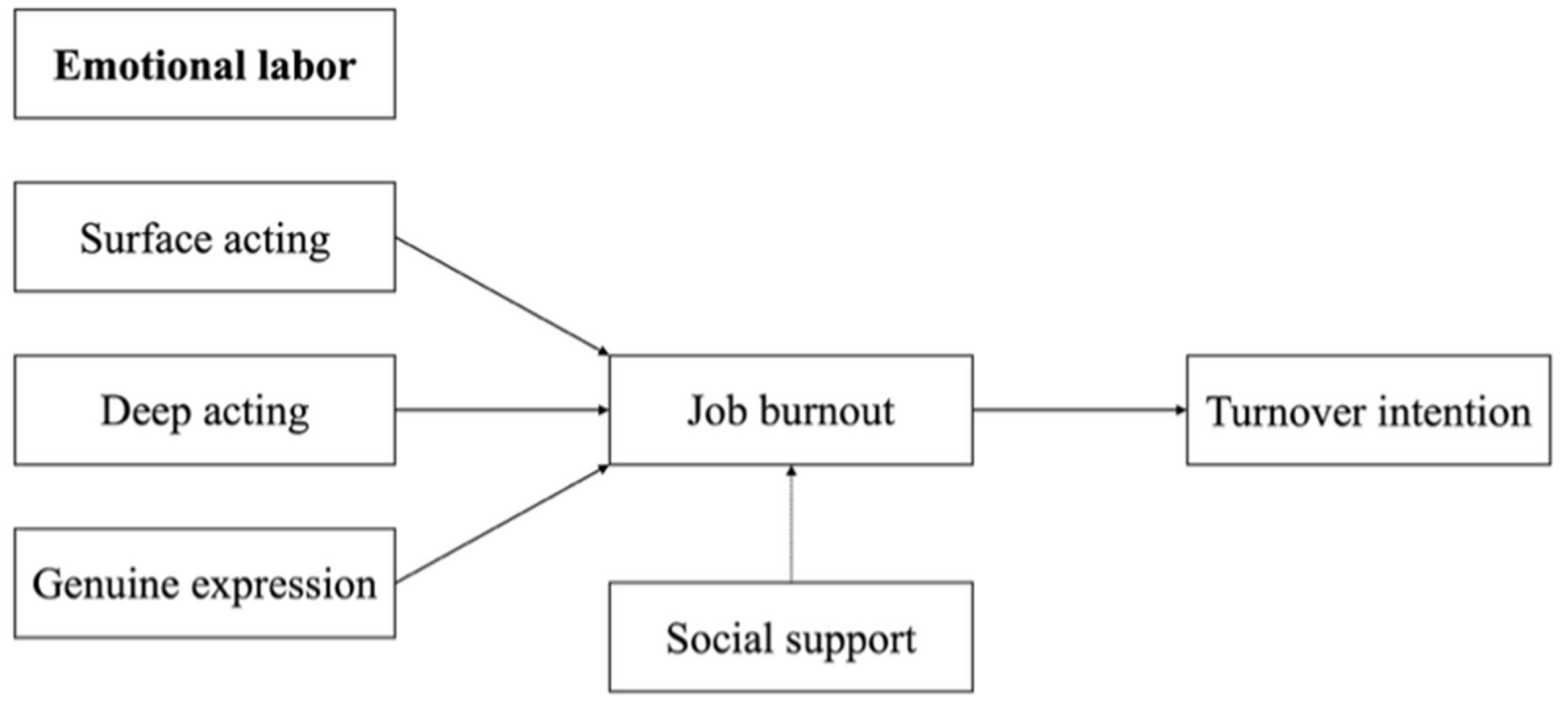
Hypothesized conceptual model based on COR theory.

This framework not only extends the applicability of emotional labor theory within educational settings but also offers a theoretical foundation for improving teacher wellbeing and designing targeted policy interventions to support rural educators. Based on the COR theory, this study proposes a conceptual framework illustrating the mediating role of job burnout and the moderating role of social support (see Figure 1).

### Job burnout: a mediating mechanism of resource depletion in emotional labor

Job burnout is a psychological condition that results from prolonged occupational stress and is characterized by three core dimensions: emotional exhaustion, depersonalization, and a diminished sense of personal accomplishment (Maslach et al., 2001). Emotional exhaustion refers to the excessive depletion of emotional and physical energy, reducing one’s work capacity. Depersonalization is defined as the development of a detached or cynical attitude toward students or other service recipients. Finally, reduced personal accomplishment reflects an individual’s sense of diminished efficacy and value in their work. In recent years, the mechanisms through which emotional labor strategies contribute to job burnout have received significant scholarly attention.

Emotional labor has been identified as a key antecedent of job burnout, with its effects varying depending on the specific emotional labor strategies employed. Surface acting, which involves adjusting outward emotional expressions to conform to external expectations without altering internal emotions, is strongly associated with emotional dissonance. Emotional dissonance—arising from the conflict between external displays and internal feelings—intensifies emotional exhaustion and contributes to job burnout (Grandey and Gabriel, 2015). According to the COR theory, surface acting continuously depletes emotional resources, and when these resources are not adequately replenished, cumulative stress leads to burnout (Hobfoll, 1989).

In contrast, deep acting involves cognitive restructuring and emotional regulation to align internal emotions with external expectations. While this strategy may alleviate emotional dissonance and enhance job satisfaction, it places high demands on emotional and cognitive resources. Over time, these demands can exacerbate resource depletion, particularly in environments with insufficient social support (Brotheridge and Grandey, 2002).

Genuine expression, on the other hand, represents to the natural alignment of external emotional expressions with internal feelings. Compared to surface and deep acting, genuine expression is less resource-intensive and has been linked to higher job satisfaction and better mental health outcomes. However, the effective implementation of this strategy depends on a supportive work environment and a strong sense of professional identity—conditions that are often lacking in resource-constrained rural educational settings.

Rural PE teachers appear particularly vulnerable to job burnout due to the interplay of multiple stressors, including resource scarcity, outdated facilities, and heavy teaching loads. These factors deplete teachers’ internal psychological resources and foster negative emotions. Moreover, the marginalization of PE as a discipline and limited career advancement opportunities reduce job satisfaction, compounding the risk of burnout. This issue is particularly pronounced in physical education, where teaching outcomes are often difficult to quantify and assess. Additionally, the phenomenon of “left-behind children”—children whose parents have migrated for work—further heightens teachers’ emotional burden. These children often experience academic, emotional, and behavioral difficulties, increasing the emotional labor required from teachers. The lack of familial support compounds teachers’ feelings of powerlessness, intensifying emotional exhaustion and fostering depersonalization.

Unmet basic psychological needs may also contribute to job burnout. In particular, teachers require a sense of autonomy, competence, and connection with others to sustain intrinsic motivation and maintain emotional balance (Deci and Ryan, 2000). For example, insufficient administrative and instructional support can undermine a sense of autonomy; ongoing challenges with student performance may weaken perceived competence; and geographical isolation, combined with limited collegial and community support, can hinder the development of meaningful interpersonal connections. The persistent failure to satisfy these needs may accelerate emotional exhaustion and reinforce the risk of burnout among rural PE teachers.

This study, grounded in COR theory, systematically examines how different emotional labor strategies contribute to job burnout in resource-scarce environments. It also explores whether genuine expression can serve as a protective factor by preserving teachers’ emotional and psychological resources.

Based on the preceding discussion, this study proposes the following hypothesis:

*H1*: Surface acting and deep acting are positively correlated with job burnout, while genuine expression is negatively correlated with job burnout.

### Turnover intention: a behavioral outcome of resource depletion

In recent years, rural schools in China have faced significant challenges, primarily due to teacher shortages and high turnover rates. Turnover intention, defined as an individual’s psychological desire to leave their current job or transition to another profession, has emerged as a critical issue for the sustainability of rural education (Tett and Meyer, 1993). Among rural PE teachers, turnover intention often manifests as aspirations to transfer to urban schools through competitive selection processes or to leave the teaching profession entirely. The primary drivers of turnover intention include limited career development opportunities, disparities in quality of life, and job burnout caused by emotional labor.

Limited opportunities for professional advancement remain a major contributor to turnover intention among rural PE teachers. These teachers face significant barriers to professional growth, particularly due to the historical marginalization of physical education as a subject. The lack of supportive policies and inadequate resource allocation further exacerbate career stagnation. Research has shown that perceived career stagnation, or the absence of advancement opportunities, significantly increases turnover intention (Ng and Feldman, 2010). From the perspective of COR theory, the conflict between the emotional demands of teaching and the limited prospects for career advancement intensifies resource depletion, thereby increasing psychological stress and turnover intention (Hobfoll, 1989). Recent findings also emphasize that career stagnation moderates the relationship between emotional labor and turnover intention, such that teachers with limited resources are more likely to develop turnover intention under high emotional labor demands (Cheung et al., 2011).

Disparities in quality of life between rural and urban areas also play a significant role in increasing turnover intention. Rural regions often lack adequate infrastructure, healthcare, and cultural amenities, all of which negatively impact teachers’ wellbeing and job stability. Furthermore, the prolonged separation of rural teachers from their families, often driven by work obligations or dissatisfaction with their children’s educational opportunities, exacerbates their psychological burden. These disparities erode teachers’ sense of belonging and emotional investment in their work, thereby accelerating job burnout and turnover intention (Li et al., 2022).

In the context of emotional labor, the formation of turnover intention is particularly complex. Surface acting, which involves significant external emotional regulation, has been strongly linked to emotional resource depletion and is a well-established predictor of turnover intention (Gabriel et al., 2015; Shin, 2012). Although deep acting can reduce emotional dissonance to some extent, its high emotional and cognitive demands, particularly in resource-scarce rural environments, may still lead to turnover intention over time (Brotheridge and Lee, 2003). Genuine expression, in contrast, which aligns external displays with internal feelings, consumes fewer emotional resources and has been associated with lower turnover intention by reducing emotional dissonance. However, the feasibility of genuine expression in rural educational environments is often constrained by external factors, such as insufficient organizational support and a lack of professional recognition, which limit its protective effects.

Job burnout is a critical mediator between emotional labor and turnover intention. Prolonged emotional resource depletion decreases job satisfaction and organizational commitment, significantly increasing turnover intention (Lee and Ashforth, 1996; Swider and Zimmerman, 2010). Among the three emotional labor strategies, the mediating role of burnout is most pronounced in the relationship between surface acting and turnover intention, as surface acting intensifies emotional exhaustion and depersonalization. These findings underscore that job burnout is not only the outcome of emotional labor imbalance but also reflects inadequate organizational support and inequitable resource distribution.

Based on the above discussion, this study proposes the following hypotheses:

*H2*: Surface acting and deep acting are positively correlated with turnover intention, while genuine expression is negatively correlated with turnover intention.

*H3*: Job burnout is positively correlated with turnover intention.

*H4*: Job burnout mediates the relationship between emotional labor and turnover intention.

### Social support: an external moderator buffering the resource depletion of surface acting

Social support refers to the emotional, informational, or tangible resources individuals receive through social interactions, which foster a sense of being cared for, accepted, and valued (Cohen and Wills, 1985). Within the framework of COR theory, social support is a critical external resource that mitigates resource depletion, strengthens emotional and psychological resilience, and reduces the negative impact of stressors on mental health. This perspective highlights the moderating role of social support in the relationship between emotional labor and job burnout.

Social support is widely recognized in organizational behavior research as a key factor in alleviating work-related stress, enhancing job satisfaction, and reducing job burnout. By providing emotional comfort and practical assistance, social support helps individuals replenish the emotional resources drained by emotional labor, thereby alleviating emotional exhaustion (Moon et al., 2013; Wen et al., 2019). For example, emotional support from colleagues not only validates and provides understanding but also enhances teachers’ sense of control by offering coping strategies for stress (García-Arroyo and Segovia, 2019). Furthermore, managerial support and recognition improve teachers’ professional identity, which in turn reduces burnout and turnover intention (Khan et al., 2020).

In rural Chinese schools, social support plays a particularly vital role but often remains inadequate due to systemic constraints and conservative management practices. While rural teachers often maintain close interpersonal relationships, the support they receive is largely emotional and lacks systemic career development initiatives or adequate resource allocation (Yin et al., 2019). Additionally, the conservative management philosophy prevalent in rural schools often undervalues physical education, further marginalizing PE teachers. Limited training opportunities, inadequate performance-based rewards, and a lack of promotion pathways diminish their professional self-esteem and sense of belonging. These factors increase their feelings of isolation and helplessness when coping with emotional labor demands. Furthermore, rural PE teachers face heightened emotional labor demands due to the academic and life challenges of rural students, compounded by scarce career aspirations and multiple stressors. These challenges intensify emotional exhaustion compared to their urban counterparts.

Research has shown that social support effectively moderates the adverse effects of emotional labor on job burnout (Cohen and Wills, 1985; Hobfoll, 1989). This moderating effect operates through two primary mechanisms. First, emotional support enhances psychological resilience, enabling teachers to recover more quickly from emotional resource depletion. Second, instrumental support offers practical solutions to workplace challenges, reducing feelings of helplessness. The impact of social support varies depending on the emotional labor strategy employed. For teachers engaging in surface acting, social support alleviates the stress caused by emotional exhaustion. For those relying on deep acting, it facilitates better alignment between internal and external emotions, thereby mitigating the negative effects of emotional labor (Asumah et al., 2019).

Based on the above discussion, this study proposes the following hypothesis:

*H5*: Social support significantly moderates the impact of emotional labor on job burnout, such that higher levels of social support reduce the negative effects of emotional labor on job burnout.

## Materials and methods

### Participants

In this study, a convenience sampling method was employed to recruit primary and secondary school PE teachers from rural areas in China. As the study targeted rural teachers rather than urban populations, several logistical and practical challenges hindered the implementation of a fully randomized sampling approach. These challenges included limited access to schools, inconsistent administrative cooperation, and infrastructural barriers in remote communities. To ensure feasibility while enhancing sample representativeness, participants were selected from rural schools across 10 geographically and socioeconomically diverse provinces—Gansu, Yunnan, Guizhou, Chongqing, Henan, Liaoning, Anhui, Shandong, Guangdong, and Jiangxi—spanning eastern, central, and western regions of China. This wide regional coverage helped mitigate potential sampling bias.

A total of 1,895 PE teachers completed an online questionnaire survey. After excluding invalid responses, 1,761 valid questionnaires were retained, resulting in an effective response rate of 92.9%. Descriptive statistics of the sample revealed that 69.56% of respondents were male, while 30.44% were female. In terms of age distribution, 30.15% of participants were between 31 and 40 years old. The majority of teachers (85.58%) were married, and 80.98% had attained at least an undergraduate degree. Regarding teaching assignments, 72.35% of respondents taught primary school students, and 51.22% exclusively taught physical education. Additionally, 37.88% reported teaching 16 to 20 PE lessons per week.

### Instruments

#### Teachers’ Emotional Labor Strategies Scale

The present study utilized the Teachers’ Emotional Labor Strategies Scale, originally developed by Diefendorff et al. (2005) and later adapted by Yin (2012). The scale comprises 13 items divided into three dimensions: surface acting, deep acting, and genuine expression. The surface acting dimension includes six items (e.g., “I need to put on airs in order to treat students or their parents in an appropriate way”). The deep acting dimension consists of four items (e.g., “I try to really feel those emotions that I have to show to my students or their parents”). The genuine expression dimension contains three items (e.g., “The emotions I show to students or their parents come naturally from within me”). A 5-point Likert-type scale was used, with “1″ indicating “strongly disagree” and “5″ indicating “strongly agree,” where higher scores reflect higher levels of emotional labor. The overall reliability of the scale was 0.81, and the Cronbach’s *α* for the three subscales were 0.82, 0.74, and 0.77, respectively, indicating good reliability.

#### Social Support Questionnaire

The Social Support Questionnaire, which was developed by Van Dick and Wagner (2001) and subsequently localized and revised by Fang and Yan (2004), was utilized in this study to assess the perceived level of social support among primary and secondary school PE teachers. The scale comprises a total of 12 question items, statements such as “School leaders and supervisors often give me good advice and guidance for my work.” A 5-point Likert-type scale was used, with “1” denoting “completely disagree” and “5” signifying “completely agree,” with higher scores indicating higher levels of perceived social support. The Cronbach’s *α* for the questionnaire in this study was 0.93, demonstrating excellent reliability.

#### Teacher Burnout Questionnaire

The Teacher Burnout Questionnaire, which was developed by Maslach et al. (2001) and subsequently localized and revised by Xinchun et al. (2016), was employed in this study to assess the extent of burnout among elementary and middle school PE teachers. The scale includes 22 items categorized into three dimensions: eight items of emotional exhaustion (“I find being a teacher a draining job”), eight items of personal fulfillment (“I can easily create a relaxing atmosphere with my students”) and six items depersonalization (“I feel that I often treat students as inanimate objects”). Responses were collected using a 5-point Likert scale, ranging from 1 (“completely disagree”) to 5 (“completely agree”). For this study, the personal fulfillment dimension was reverse scored. The Cronbach’s *α* of the total scale in this study was 0.86, with individual Cronbach’s α for the three subscales of 0.94, 0.94, and 0.89, respectively, indicating high internal consistency.

#### Intention to Leave Scale

The present study utilized Bradley’s Intention to Leave Scale (Bradley, 2007) to assess the intention to resign among primary and secondary PE teachers. The scale comprises three items, such as, “I have recently wanted to engage in a different, new career,” employing a 5-point Likert-type scale, with “1” denoting “strongly disagree” and “5” signifying “strongly agree.” The scale is scored on a 5-point Likert scale, with “1” indicating “strongly disagree” and “5” indicating “strongly agree,” with higher scores indicating stronger intention to leave. The Cronbach’s α of the scale in this study was 0.89, indicating high internal consistency.

#### Quality control

Data collection for this study was conducted between March 15 and July 10, 2024. During this period, the research team contacted school administrators and local education authorities, who assisted in distributing the survey link and QR code to PE teachers in rural primary and secondary schools. Given the wide geographic distribution of the target population, data were collected online to enhance accessibility and participation.

Participation in the study was entirely voluntary, and all respondents remained anonymous. Before completing the questionnaire, participants were informed of the purpose and procedures of the study, and assured that their data would be kept strictly confidential and used solely for research purposes. Submission of the completed questionnaire was considered as provision of informed consent. No personally identifiable information was collected, and all data were securely stored and managed.

This study received ethical approval from the Ethics Committee of Capital University of Physical Education and Sports (Approval No. 2024A033), and research permission was obtained from the participating institutions.

#### Data processing and statistical analysis approach

Descriptive statistics and correlation analyses for the target variables were conducted using SPSS 29.0. Structural equation modeling (SEM) was first performed using AMOS 26.0 to examine the overall model fit and relationships among the latent constructs. Additionally, Model 7 in the PROCESS macro was employed to test the moderating effect of social support on the mediating pathway “emotional labor → burnout → turnover intention.” Parameter estimates were obtained using the bias-corrected bootstrap method with 5,000 resamples, and 95% confidence intervals were reported to determine the significance of the effects. Variance inflation factor (VIF) values were examined to assess multicollinearity, with all values below 5, indicating an acceptable level of collinearity among predictors.

## Results

### Common method bias testing

Harman’s one-factor test was employed to evaluate the potential presence of common method bias in this study. An exploratory factor analysis (EFA) was conducted on all key variables, including surface acting, deep acting, genuine expression, social support, burnout, and turnover intention. The results indicated that the variance explained by the first principal component was 27.5%, which is below the critical threshold of 40%. This finding suggests that common method bias is unlikely to be a significant concern in this study.

### Descriptive statistics and correlation

To improve the transparency of data reporting and assess the appropriateness of the statistical analyses, we examined the distributional characteristics of all key variables, including measures of central tendency, dispersion, and normality indices (i.e., skewness and kurtosis). The results showed that all core variables had skewness values ranging from −0.704 to 0.608 and kurtosis values ranging from −0.417 to 1.413. These values fall within the commonly accepted thresholds (|skewness| < 1 and kurtosis < 3), indicating that the distributions of the variables approximate normality.

Specifically, variables such as burnout (skewness = −0.416, kurtosis = 0.096), deep acting (skewness = −0.475, kurtosis = 0.877), and genuine expression (skewness = −0.704, kurtosis = 1.120) exhibited slight negative skewness but remained within acceptable bounds. Surface acting and turnover intention showed modest positive skewness (0.287 and 0.608, respectively), and social support demonstrated a high but acceptable kurtosis of 1.413, suggesting mild peakedness. Overall, the data satisfied the assumption of approximate normality, supporting the use of parametric statistical methods such as regression and mediation analysis.

Table 1 summarizes the descriptive statistics for each variable, including the mean (M), standard deviation (SD), and Pearson’s correlation coefficients. The results indicate that surface acting and deep acting are significantly positively correlated with both burnout and turnover intention. In contrast, genuine expression is significantly negatively correlated with burnout and turnover intention. Additionally, social support is significantly negatively correlated with surface acting, burnout, and turnover intention, while showing significant positive correlations with both deep acting and genuine expression.

**Table 1:** Descriptives statistics and correlations.

Variables	*M*	*SD*	1	2	3	4	5	6
1. SA	2.50	0.81	1					
2. DA	3.29	0.74	0.41^**^	1				
3. GE	3.64	0.75	−0.13^**^	0.43^**^	1			
4. SS	3.79	0.62	−0.25^**^	0.08^**^	0.30^**^	1		
5. Burnout	2.43	0.56	0.44^**^	0.08^**^	−0.20^**^	−0.51^**^	1	
6. TI	2.27	1.01	0.34^**^	0.05^*^	−0.14^**^	−0.38^**^	0.57^**^	1

**p < 0.01. SA, surface acting; DA, deep acting; GE, genuine expression; SS, social support; TI, turnover intention.

### Simple mediation effect test

Structural equation modeling was conducted using AMOS 26.0 to examine the simple mediating effects of burnout in the relationship between emotional labor and turnover intention.

The structural equation model examining the mediating effect of burnout between surface acting and turnover intention showed acceptable fit: RMSEA = 0.065; SRMR = 0.024; CFI = 0.982; TLI = 0.969; GFI = 0.984; AGFI = 0.962. Surface acting significantly predicted turnover intention both directly (*β* = 0.30, *p* < 0.001) and indirectly through job burnout. When burnout was included as a mediator, the direct effect remained significant (*β* = 0.11, *p* < 0.001), and the indirect effect was 0.30 (95% CI [0.26, 0.34]), accounting for 69% of the total effect (see Table 2).

**Table 2:** Analysis of the mediating effect of job burnout on emotional labor and turnover intention.

Path	Variable	Predictor	*β*	*SE*	*t*	95% CI (LLCI, ULCI)	*R*	*R^2^*	*F*
1a	Burnout	SA	0.44	0.02	20.68***	[0.40, 0.48]	0.44	0.20	427.55***
1b	TI	Burnout	0.53	0.02	24.25***	[0.48, 0.57]	0.58	0.34	444.62***
1c’	TI	SA	0.11	0.02	4.84***	[0.06, 0.15]			
2a	Burnout	DA	0.08	0.02	3.44***	[0.04, 0.13]	0.08	0.01	11.83***
2b	TI	Burnout	0.57	0.02	29.12***	[0.53, 0.61]	0.57	0.33	427.25***
2c’	TI	DA	0.01	0.02	0.19	[−0.04, 0.04]			
3a	Burnout	GE	−0.20	0.02	−8.61***	[−0.25, −0.16]	0.20	0.04	74.19***
3b	TI	Burnout	0.57	0.02	28.38***	[0.53, 0.61]	0.57	0.33	428.6***
3c’	TI	GE	−0.03	0.02	−1.36	[−0.01, 0.01]			

Path labels (e.g., 1a, 1b, 1c’) refer to components of the mediation model structure. Path c’ represents the direct effect. ^*^p<0.05, ^**^p < 0.01, ^***^p < 0.001.

The structural equation model examining the mediating effect of burnout between deep acting and turnover intention showed acceptable fit: RMSEA = 0.080; SRMR = 0.026; CFI = 0.971; TLI = 0.949; GFI = 0.976; AGFI = 0.943. For deep acting, the direct effect on turnover intention was not significant (*β* = 0.01, *p* > 0.05), but it negatively predicted job burnout (*β* = 0.08, *p* < 0.001), which in turn significantly predicted turnover intention (*β* = 0.57, *p* < 0.001). The indirect effect was 0.064 (95% CI [0.02, 0.11]), accounting for 93% of the total effect (see Table 2).

The structural equation model examining the mediating effect of burnout between genuine expression and turnover intention showed acceptable fit: RMSEA = 0.095; SRMR = 0.032; CFI = 0.961; TLI = 0.932; GFI = 0.968; AGFI = 0.925. Similarly, the direct effect of genuine expression on turnover intention was non-significant (*β* = −0.03, *p* > 0.05), while its negative association with job burnout (*β* = −0.20, *p* < 0.001) indirectly reduced turnover intention (*β* = 0.57, *p* < 0.001). The indirect effect was −0.114 (95% CI [−0.147, −0.081]), accounting for 81.4% of the total effect (see Table 2).

### Moderated mediated effects

Controlling for gender, age, and teaching tenure, regression results (see Table 3) showed that surface acting significantly predicted higher job burnout (*β* = 0.23, *p* < 0.001), while social support significantly predicted lower burnout (*β* = −0.38, *p* < 0.001). The interaction between surface acting and social support was also significant (*β* = 0.06, *p* = 0.001; 95% CI [0.02, 0.09]).

**Table 3:** The moderating role of social support in surface acting and job burnout.

Predictor	*β*	*p*	95% CI	*R* ^2^
SA	0.23	< 0.001	–	0.374
SS	−0.38	< 0.001	–	
SA × SS	0.06	0.001	[0.02, 0.09]	
DA	0.1	< 0.001	–	0.276
SS	−0.47	< 0.001	–	
DA × SS	−0.02	0.382	[−0.06, 0.02]	
GE	−0.04	< 0.001	–	0.263
SS	−0.44	< 0.001	–	
GE × SS	0.002	0.897	[−0.03, 0.04]	

SA, surface acting; DA, deep acting; GE, genuine expression; SS, social support; TI, turnover intention.

For deep acting, it significantly predicted higher burnout (*β* = 0.10, *p* < 0.001), and social support remained a significant negative predictor (*β* = −0.47, *p* < 0.001). However, the interaction term between deep acting and social support was not significant (*β* = −0.02, *p* = 0.382; 95% CI [−0.06, 0.02]).

For genuine expression, both the predictor and social support were significant negative predictors of burnout (*β* = −0.04 and *β* = −0.44, respectively; both *p* < 0.001). However, their interaction was not significant (*β* = 0.002, *p* = 0.897; 95% CI [−0.03, 0.04]).

**Figure 2:**
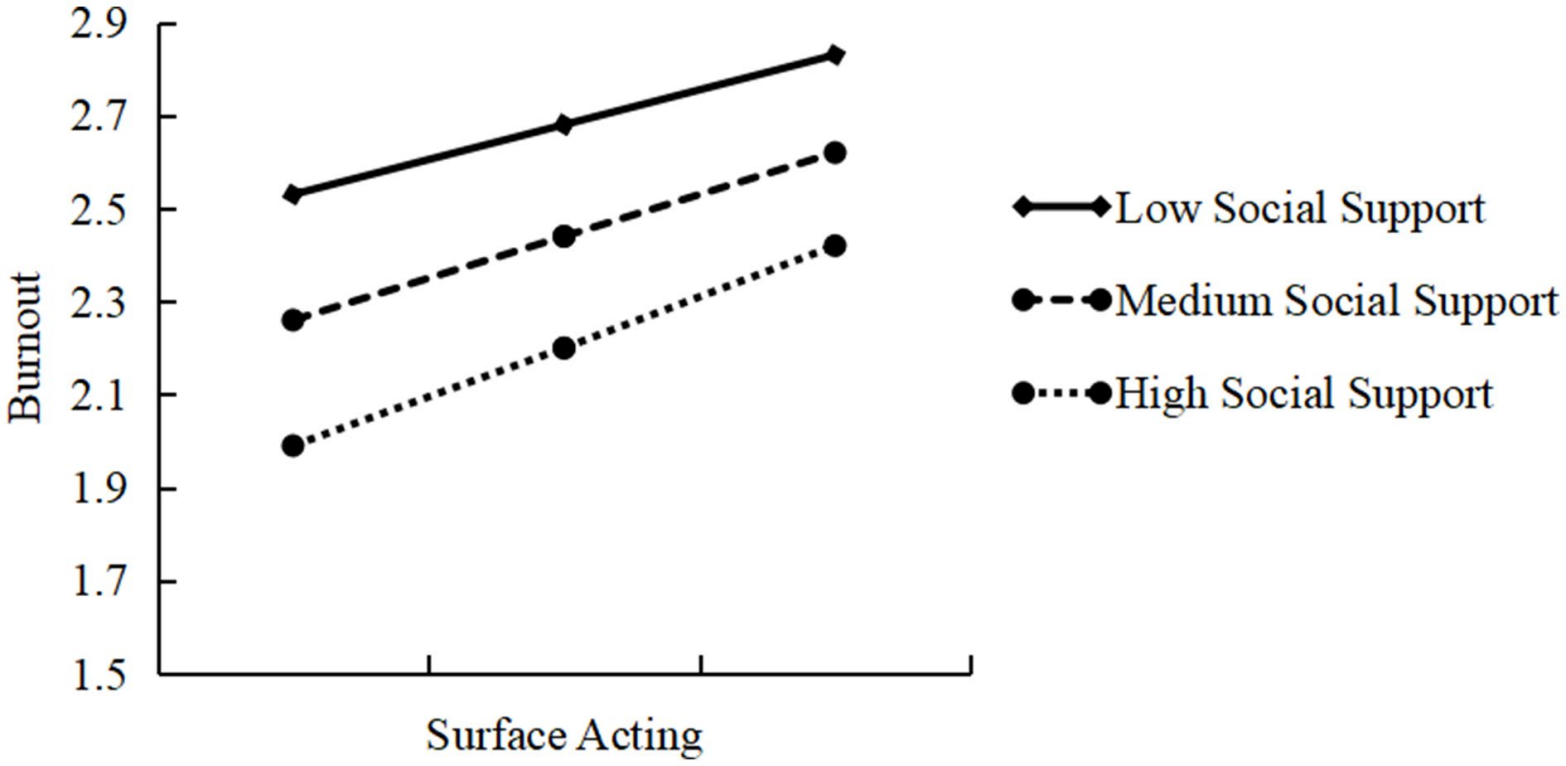
The moderating effect of social support on surface acting and job burnout.

To further illustrate the moderating effect of social support on the relationship between surface acting and job burnout, a simple slope analysis was conducted. Social support was categorized into high and low groups, based on values one standard deviation above and below the mean, respectively. The results, as shown in Figure 2, indicate that the impact of surface acting on job burnout depends significantly on the level of social support. Specifically, when social support was low, surface acting exhibited a stronger positive association with job burnout. In contrast, when social support was high, the relationship between surface acting and job burnout was weaker, suggesting that high social support buffers the negative effects of surface acting.

## Discussion

Grounded in Conservation of Resources (COR) theory, this study examined how emotional labor strategies influence turnover intention among rural PE teachers through the mediating role of job burnout and the moderating role of social support. The findings reveal that emotional labor in resource-constrained rural educational settings not only reflects individual coping strategies but also highlights the structural pressures placed on teachers’ psychological resources. The following discussion explores the psychological effects of the three emotional labor strategies, the mediating mechanism of job burnout, and the boundary conditions of social support.

First, the study found that surface acting not only directly predicted turnover intention but also exerted a significant indirect effect via job burnout. This indicates that emotional resource depletion serves as a critical transmission mechanism linking surface acting to teachers’ intention to leave. Because surface acting entails a dissonance between outward behavior and inner feelings, it often leads to emotional strain and cognitive fatigue (Brotheridge and Grandey, 2002). These effects are particularly intensified in rural contexts, where PE teachers face marginal professional status and limited institutional feedback. As such, the prolonged use of surface acting becomes a default coping pattern that triggers a psychological spiral of “inauthentic display → resource exhaustion → professional alienation,” consistent with COR theory’s “resource loss spiral” (Hobfoll et al., 2018). Hence, in rural education, surface acting should not be viewed as a temporary adjustment but as a problematic emotional survival mechanism that warrants serious attention from educational administrators.

Second, regarding deep acting, the study found no significant direct effect on turnover intention, but a significant indirect effect through burnout. This suggests that even when teachers attempt to align internal emotions with professional demands through deep emotional regulation, the cognitive and emotional effort required may not be adequately compensated in under-resourced environments. Instead, the high investment–low return dynamic may further intensify psychological strain. This finding aligns with Hülsheger and Schewe (2011), who noted that in contexts lacking professional development and emotional support systems, the adaptive value of deep acting may diminish over time. Therefore, if deep acting is universally regarded as a beneficial strategy, it overlooks its contextual dependency and potential risks under conditions of unequal resource distribution.

Third, genuine expression was shown to reduce turnover intention indirectly by decreasing job burnout. As a low-resource emotional labor strategy, genuine expression enhances emotional congruence and improves teacher-student relationships, thereby increasing job satisfaction and psychological resilience (De Ruiter et al., 2021). However, its effectiveness relies on the presence of strong professional identity, psychological safety, and organizational trust—conditions often unmet in rural schools. Therefore, the actual applicability of this strategy in such settings may be overstated. Rather than promoting genuine expression as a personal virtue, educational reform should prioritize building supportive organizational environments that enable genuine expression to occur sustainably.

Additionally, this study confirmed that job burnout plays a crucial mediating role between emotional labor and turnover intention. The findings support COR theory’s proposition that sustained resource depletion leads to behavioral withdrawal. Turnover intention, therefore, is not simply a direct result of emotional labor but emerges from prolonged psychological strain (Swider and Zimmerman, 2010). Nonetheless, it is important to acknowledge that due to the study’s cross-sectional design, causal relationships among variables cannot be definitively established. Reverse causality remains a possibility—for instance, teachers experiencing higher burnout may be more prone to emotional labor, or those with stronger turnover intentions may perceive emotional demands more acutely. Future research should employ longitudinal or mixed-method designs to further test the temporal sequencing of these pathways.

Finally, the moderating role of social support was significant only in the path from surface acting to job burnout. Specifically, under low levels of social support, surface acting had a stronger negative impact on burnout, whereas high levels of support buffered this effect. In contrast, social support showed no significant moderating effects in the paths involving deep acting or genuine expression. This finding holds both theoretical and practical implications. Surface acting triggers pronounced emotional dissonance, which may be more easily addressed through external emotional or instrumental support. By contrast, deep acting and genuine expression rely more on internalized motivation and long-term professional socialization, and may not respond as readily to short-term support interventions. This highlights the need for tailored support strategies that recognize the differing resource profiles and demands of each emotional labor strategy, rather than applying uniform interventions.

Taken together, this study not only validates the applicability of COR theory in educational contexts but also reveals the psychological costs and adaptive boundaries of various emotional labor strategies. The findings emphasize that emotional labor in resource-poor environments is highly contextual and should not be treated merely as an individual regulatory task. Future research should integrate qualitative or mixed-method approaches to further explore how teachers perceive and enact emotional labor. Policymakers are encouraged to adopt a systemic intervention framework that combines structural support, organizational empowerment, and emotional relief to enhance teacher wellbeing and ensure the long-term sustainability of the rural education workforce.

### Implications of the study

The findings of this study offer multidimensional practical implications for improving the professional wellbeing of rural PE teachers, mitigating their turnover intentions, and optimizing educational governance—especially in resource-constrained settings. They are particularly relevant to advancing the goal of educational equity outlined in SDG4, by highlighting how targeted emotional labor interventions can contribute to systemic change.

First, teacher professional development systems in rural areas should explicitly incorporate psychological wellbeing and emotional labor competence as core components. Traditional training programs often emphasize instructional techniques while neglecting emotional regulation skills. This study recommends the integration of evidence-based psychological interventions, such as cognitive restructuring training, mindfulness-based stress reduction sessions, and emotion recognition skill modules. These interventions are practical, low-cost, and well-suited for rural implementation through support from teacher education colleges and online delivery platforms. Importantly, they can improve teachers’ emotional resilience and self-regulation without imposing significant financial burdens on local education budgets.

Second, school administrators can build teacher support systems through organizational innovation, even when resources are limited. We propose two actionable strategies:

Tiered Mentorship Models: Schools can implement a “dual mentorship system,” where one experienced senior teacher provides subject-specific and pedagogical guidance, while an administrative mentor (e.g., the academic dean) offers institutional support. This “professional + structural” approach helps compensate for external resource scarcity by reorganizing and reallocating internal human resources in a cost-effective manner.

Embedded Teacher Support Networks: Schools can establish peer-support groups, topic-based learning communities, or designated emotional expression spaces (e.g., “emotion corners”) that serve as platforms for routine communication, emotional relief, and collective identity formation. In remote regions, initiatives such as peer mentoring between veteran and novice teachers and the formation of small-scale “micro-communities” can foster horizontal emotional support, improving the overall quality of interpersonal connections among teachers.

Furthermore, policymakers should ensure that financial investments are precisely targeted and coupled with institutional safeguards. In addition to improving salaries and employment security, we recommend the implementation of a “teaching burden reduction list,” which explicitly defines and limits non-instructional administrative responsibilities assigned to teachers. Such mechanisms can prevent role overload and burnout. Additionally, a linkage mechanism between performance evaluation and psychological wellbeing is advisable—incorporating teacher wellbeing indicators into school accountability frameworks can compel school leaders to create more supportive environments. Compared with large-scale infrastructure projects, these institutional interventions are more cost-effective and sustainable under tight fiscal constraints.

Finally, while aligning national policies with global agendas such as SDG4, it is essential to respond to culturally specific tensions in the Chinese context. In traditional culture, emotional suppression and silent endurance are often valorized as teacher virtues. This may cause teachers to internalize stress, avoid seeking help, and conceal burnout symptoms. This study highlights the need for educational leaders to move beyond the cultural logic of “individual resilience” and foster an organizational ethos that recognizes emotional labor as a collective and institutional responsibility.

### Limitations of the study

While this study provides empirical evidence for the mechanisms through which emotional labor influences job burnout and turnover intention among rural PE teachers—and contributes both theoretically and practically—it also presents several limitations that should be addressed in future research.

First, the use of a cross-sectional survey design limits the ability to establish causal relationships or capture dynamic processes among variables. Resource depletion and behavioral responses induced by emotional labor are typically cumulative in nature and may involve time-lagged effects. Future research should consider adopting longitudinal tracking designs to examine how teachers’ emotional labor strategies and psychological states evolve over time, thereby providing a more accurate understanding of the causal chains linking emotional labor, burnout, and turnover intention.

Second, the study relied exclusively on self-reported questionnaire data, which may introduce common method bias and social desirability effects. The limitations of survey-only approaches have been increasingly criticized in educational psychology. Scholars have pointed out that single-source quantitative surveys may fail to capture the complexity of psychological processes, highlighting the need to complement such data with qualitative methods (Al-Hoorie et al., 2024). Due to practical constraints, this study was unable to incorporate interviews or observational data. However, we acknowledge that a mono-method design may underestimate the richness of teachers’ subjective experiences. Future studies are encouraged to adopt mixed-method designs, integrating third-party assessments (e.g., ratings from colleagues or school administrators), classroom observations, or physiological indicators (e.g., cortisol levels, heart rate variability) to improve measurement validity and interpretive depth. Additionally, statistical approaches such as the marker variable technique could be used to statistically control for potential common method variance.

Third, the sample was limited to rural PE teachers in China, which restricts the generalizability of the findings. The impact of emotional labor is likely influenced by regional disparities in resource allocation, disciplinary status, and institutional governance. For example, the marginalization of PE as a subject, access to social support networks, and teachers’ psychological resilience may vary significantly across contexts. Furthermore, cultural values—such as emotional restraint and self-sacrificial professionalism—are prevalent in the Chinese educational context and may shape how teachers perceive emotional labor and tolerate psychological strain. Future research should examine the moderating role of cultural factors in the relationship between emotional expression strategies and psychological outcomes. Comparative studies across disciplines (e.g., language arts, science, the arts) and rural teacher populations in other socio-cultural contexts would also help identify both generalizable patterns and culturally specific dynamics of emotional labor.

## Conclusion

Grounded in COR theory, this study systematically examined the impact of emotional labor strategies on turnover intention among rural PE teachers, highlighting the mediating role of job burnout and the moderating role of social support. The findings indicate that surface acting significantly increases turnover intention by intensifying job burnout; deep acting does not directly predict turnover intention but exerts an indirect effect through burnout; and genuine expression, as a low-resource strategy, helps alleviate burnout and reduce the risk of turnover. Moreover, social support buffers the negative impact of surface acting on burnout, though it shows no significant moderating effect in the deep acting or genuine expression pathways.

These results extend the applicability of emotional labor theory to under-resourced educational contexts and underscore the importance of strategy-specific and context-sensitive approaches to emotional labor. The study calls for education administrators and policymakers to strengthen structural support for teachers’ emotional labor and foster emotionally supportive organizational cultures to enhance the wellbeing of rural teachers and promote the long-term sustainability of the education system.

## Data Availability

The original contributions presented in the study are included in the article/supplementary material, further inquiries can be directed to the corresponding author.
